# Ecotoxicological Evaluation of the Antimicrobial Butylparaben in Edaphic Organisms Using Multiple Biomarkers

**DOI:** 10.1002/tox.24568

**Published:** 2025-09-20

**Authors:** Lorena Maihury Santos Tsubouchi, Edson Araújo de Almeida, Diane Scapin, Anna Karolina Gomes Oliveira, Cassiano Aparecido de Souza, Diego Espirito Santo, Carmem Lúcia Henrich, Ana Elisa Maehashi, Gideã Taques Tractz, Craig Allan Downs, Osvaldo Valarini Junior, Regiane da Silva Gonzalez, Elisângela Düsman, Ana Paula Peron

**Affiliations:** ^1^ Postgraduate Program in Chemical Engineering, Polytechnic Institute of Bragança Bragança Portugal; ^2^ Postgraduate Program in Chemistry Maringá State University Maringá Paraná Brazil; ^3^ Postgraduate Program in Environmental Engineering Federal Technological University of Paraná Francisco Beltrão Paraná Brazil; ^4^ Postgraduate Program in Environmental Engineering and Technology Federal University of Paraná Palotina Paraná Brazil; ^5^ Environmental and Sanitary Engineering Course Federal Technological University of Paraná Paraná Brazil; ^6^ Chemical Engineering Course Federal Technological University of Paraná Paraná Brazil; ^7^ Academic Department of Chemistry Federal University of Technology of Paraná Paraná Brazil; ^8^ Haereticus Environmental Laboratory Clifford USA; ^9^ Postgraduate Program in Technological Innovations Federal Technological University of Paraná Paraná Brazil; ^10^ Postgraduate Program in Food Technology. Federal Technological University of Paraná Paraná Brazil

**Keywords:** escape, mitotic spindle alterations, mortality, oxidative stress, paraben, reduced cell division

## Abstract

Butylparaben (BuP) recurrently contaminates soils worldwide, mainly by incorporating sewage sludge into cultivated areas, using wastewater in irrigation, and leaching contaminated soils. However, there are few studies on the ecotoxicological effects of this paraben on edaphic organisms. The ecotoxicity of BuP was evaluated in seeds of *Daucus carota* , *Allium cepa* , and *Cucumis sativus* , in the roots of *A. cepa* bulbs, and in *Eisenia fetida* earthworms, at concentrations of 10, 50, 100, and 500 ng/L. In root meristems, the four concentrations of BuP induced lipid peroxidation and raised the levels of superoxide radicals, which triggered inhibition of cell division and mitotic spindle alteration, significantly reducing the growth of roots in seeds and bulbs. In animals, BuP at 10, 50, 100, and 500 ng/L caused 80%, 80%, 70%, and 90% evasion of earthworms from artificial soil, respectively. In addition, this paraben did not cause mortality in earthworms after 14 days of exposure. However, all concentrations increased the production of superoxide and hydroxyl radicals in cells and caused lipid peroxidation. Thus, increased exposure to this compound can affect the ecological functions negatively and/or cause the death of these animals. Therefore, recurrent contamination with BuP can negatively impact soil quality, posing a risk to agricultural productivity and the environment. This study is a pioneer in the ecotoxicological evaluation of BuP in plants at environmentally relevant concentrations and in the behavioral and oxidative stress study in earthworms.

## 1 Introduction

Parabens (alkyl esters of p‐hydroxybenzoic acid) have high antimicrobial activity and are widely used as preservatives by the pharmaceutical, food, cosmetics, and personal care products industries [1, 2]. Annually, more than 8000 tons of parabens are used worldwide [3, 4]. Among these compounds, butylparaben (BuP), with log Kow 3.57 and molecular weight 194.25 g/mol, is the most widely used industrially because it has good stability in aqueous media, at different pHs and temperatures, and low toxicity to humans [2].

BuP is a pollutant in the emerging class because its release into different environmental matrices has not yet been legislated. Its biodegradation in water and soil is relatively fast (on average 5 days), but its release into the environment is uninterrupted, classifying it as pseudopersistent [5]. Traditional treatment methods do not efficiently remove this paraben from wastewater, and in water resources, it is generally found in concentrations ranging from ng to μg/L^2^. In aquatic organisms, adverse effects assessment studies have shown that BuP caused acute toxicity and death in planaria [6], high mortality in microcrustaceans, and reduced luminescence in bacteria [7], as well as reduced heart rate and blood circulation, neurobehavioral toxicity, teratogenicity, endocrine disruption, and bioaccumulation in fish [5, 8, 9].

Soils around the world are recurrently contaminated with BuP, mainly due to the incorporation of biosolids into agricultural soils, the use of treated and untreated wastewater in irrigation, the discharge of effluents directly into the soil, and leaching from contaminated areas [2, 5]. This antimicrobial is found in soils, biosolids, and wastewater at concentrations in the ng/L range [10, 11, 12]. Unlike aquatic organisms, there are practically no ecotoxicological studies on BuP in edaphic organisms in the literature. The environmentally relevant studies found were by Samarasinghe et al. [1], who found that exposure to this compound did not affect growth and reproduction in earthworms, and by Nagar et al. [13], in which this paraben triggered toxicity and endocrine disruption in soil nematodes. Therefore, it is imminent to carry out further studies on the action of BuP on soil species to determine its dangerousness to this environmental matrix.

The species *Daucus carota* L. (carrot), *Allium cepa* L. (onion), and *Cucumis sativus* L. (cucumber) are standard bioassays in ecotoxicological studies of environmental contaminants, such as emerging pollutants, evaluated based on physiological and biochemical parameters [14, 15, 16, 17, 18], and recommended by the United States Environmental Protection Agency (USEPA) [19] and the Organization for Economic Cooperation and Development (OECD) [20]. *Allium cepa* L. bulb roots are a robust bioassay that has been used worldwide for more than five decades to assess the systemic and cellular toxicity of environmental pollutants and for environmental monitoring [21, 22, 23, 24]. Among their important advantages as a biological model is the high similarity of their physiological, cytological, and biochemical results with other plants, mammals, and in vitro tests [18, 21, 22, 23, 25, 26, 27, 28]. *Eisenia fetida* Sav. earthworms are an important edaphic representative and are fundamental in the terrestrial trophic chain, being widely used to assess the toxicity of environmental pollutants present in the soil [6, 17, 29, 30]. In addition to their sensitivity to chemical and physical changes in the soil, earthworms exhibit behavioral, physiological, and biochemical changes in response to contaminated soils, allowing for early detection of pollution [31, 32].

In the present study, BuP was evaluated at environmentally relevant concentrations and through multiple biomarkers for systemic and cellular toxicity in *D. carota* , *A. cepa* , and *C. sativus* plants and in *E. fetida* earthworms. The results will contribute to understanding the environmental risk of this compound in the soil and will help to build regulations that limit the disposal of parabens in the environment.

## 2 Material and Methods

### 2.1 Obtaining BuP and Defining and Preparing the Concentrations for the Study

Butylparaben (p‐hydroxybenzoic acid n‐butyl ester), CAS 94‐26‐8, was obtained in analytical grade from Sigma‐Aldrich, as were the other reagents used in this study.

Identification and quantification assessments carried out in different countries have shown that BuP has been found in soils at concentrations of 101.6–501.4 ng/L [10, 12], in sewage sludge from 27 to 107 ng/L [10, 11], and in treated wastewater from 10.1 to 50.9 ng/L^12^. Based on this research, the concentrations defined for study in plants and earthworms were 10, 50, 100, and 500 ng/L.

BuP solutions were prepared in aqueous medium with the addition of Tween 80 (at the same mass concentration as the reagent) under stirring in an Ultra‐Turrax disperser (IKA). Concentrations of 10, 50, 100, and 500 ng/L were prepared by dilutions from a stock solution of 0.03 g/L. All prepared solutions were stored in the absence of light. The preparation of BuP solutions was carried out based on the studies by Nascimento et al. [17], Okon et al. [4], and Vaz et al. [18], which evaluated the ecotoxicity of other parabens.

### 2.2 Stability Analysis of BuP in Aqueous Medium for 7 Days

A BuP stock solution of 0.03 g/L was prepared, and its stability in aqueous media was evaluated without light for 7 days (Equation 1). The analyses were carried out using a UV–Vis spectrophotometer at 255 nm. The results were presented in percentages. The number of days chosen to evaluate the stability of BuP in aqueous medium corresponds to the time in which the seeds of the different species and the onion bulbs used in the present study remained in contact with the concentrations of this compound.

(1) $$S\left(\%\right)=\frac{A_{s}}{A_{0}}\times 100$$

where *S* is the stability, *A*_s_ the absorbance of the sample, and *A*_0_ the initial absorbance on day 0.

### 2.3 Test With Plants

#### 2.3.1 Evaluation of Phytotoxicity in Seeds of *D. carota*, *A. cepa*, and *C. sativus*

Germination tests were conducted according to OECD [20]. Seeds of *D. carota* , *A. cepa* , and *C. sativus* were used, branded Isla, non‐transgenic, and free of agrochemicals. According to the information on the packaging, the seed batches had a germination rate of over 97% and a purity of between 99% and 100%. Seeds from the same batch were used throughout the experiment.

For the phytotoxicity analysis, seeds of each species were distributed in previously sterilized Petri dishes and lined with filter paper. Twenty seeds were used per plate; each treatment was analyzed in quintuplicate. The filter paper was moistened on each plate with the treatment solution (1.5 mL), taking care not to soak it. The plates were then wrapped in plastic wrap to prevent drying out and placed in a Biochemical Oxygen Demand (BOD) incubator at 25°C, where they remained for the duration of the experiment (7 days) in the absence of light. Distilled water was used as a control.

A seed was considered germinated after the emergence of the radicle. The germination percentage was calculated according to Equation (2).

(2) $$G\left(\%\right)=\frac{\mathrm{SG}}{\mathrm{TS}}\times 100$$

where *G* is the germination, SG is the number of seeds germinated, and TS is the total number of seeds used.

After 7 days, the seed roots were measured with a digital caliper, and the Relative Growth Index (RGI) was calculated (Equation 3).

(3) $$\mathrm{RGI}=\frac{\mathrm{RLI}}{\mathrm{RLC}}$$

where RGI is the Relative Growth Index, RLI is the average length of the roots exposed to the treatment, and RLC is the average length of the control roots.

According to the protocol of Biruk et al. [33], RGI values between 0.8 and 1.2 (0.8 ≤ RGI ≤ 1.2) indicate that the treatments did not affect root elongation, while values below 0.8 (0.1 < RGI < 0.8) indicate inhibition of root growth, with lethal potential, and values above 1.2 (RGI > 1.2) indicate stimulation of root growth.

The Germination Index (GI) was calculated according to Equation (4). According to Mañas and Heras [34], germination indices less than or equal to 50% (GI ≤ 50%) indicate high risk for the plant, values between 50% and 80% (50% < GI < 80%) indicate moderate risk, and values greater than or equal to 80% (GI ≥ 80%) indicate low risk.

(4) $$\mathrm{GI}\;\left(\%\right)=\frac{\mathrm{RLI}\times \mathrm{GSI}}{\mathrm{RLC}\times \mathrm{GSC}}\times 100$$

where GI is the Germination Index, RLI the average length of roots exposed to treatment, RLC the average length of roots in the control, GSI the number of seeds germinated under exposure to treatment, and GSC the number of seeds germinated in the control.

#### 2.3.2 Evaluation of Cytotoxicity and Genotoxicity in *A. cepa* Roots

The tests with *A. cepa* followed the protocol of Fiskesjö [35] with adaptations by Nascimento et al. [17]. The onions were purchased from an organic garden. The dehydrated cataphylls and dried roots were removed, and the bulbs were washed under running water. The onions were then distributed in beakers with the treatment solutions so that the root growth zone was submerged in the solution. The bulbs were placed in a BOD oven for 7 days in the dark. The treatment solutions were prepared and changed daily. Distilled water was used as a control. Five onion bulbs were used for each treatment.

After incubation, roots from each bulb were collected and fixed in Carnoy 3:1 for 24 h. After this time, slides of the meristematic regions of the roots were mounted and analyzed under an optical microscope using a 40× objective lens.

To assess cytotoxicity, 2000 cells from each bulb were analyzed for 10 000 cells per treatment, and the Mitotic Index was calculated (Equation 5). To assess genotoxicity, 200 cells from each bulb were analyzed for 2000 cells per treatment, and the Cell Alteration Index (CAI) was calculated (Equation 6). The cellular alterations considered were multipolar spindles and polyploidy, micronucleus, chromosomal abnormalities in prophase, metaphase, anaphase, and telophase, viscosity, chromosomal bridges in anaphase and telophase, and chromosomal breaks.

(5) $$\mathrm{MI}=\frac{\mathrm{DC}}{\mathrm{TC}}\times 100$$

(6) $$\mathrm{CAI}=\frac{\mathrm{AC}}{\mathrm{TC}}\times 100$$

where MI is the Mitotic Index, DC is the number of dividing cells, TC is the total number of cells analyzed, CAI is the Cell Alteration Index, and AC is the total number of cells altered.

##### 2.3.2.1 Absorption of Treatments by the Roots

For each treatment, 50 mg of roots from each bulb repetition (obtained in 2.3.2) was macerated in distilled water for 15 min at 4000 rpm, and the supernatants (homogenates) were collected. For absorption evaluation, the absorbance of the homogenates was determined at 255 nm. A curve was constructed for BuP at different concentrations, based on the range of 360 to 10 ng/mL, with an *R* ^2^ of 0.99.

### 2.4 Tests With *E. fetida*

#### 2.4.1 Obtaining the Earthworms Constitution of the Artificial Soil, and Number of Replicates for Each Treatment

The *E. fetida* earthworms were purchased from the worm farm of the Federal Technological University of Paraná, Francisco Beltrão Campus, Paraná, Brazil. Two‐month‐old earthworms with well‐developed clitellum and a body weight between 500 and 600 mg were selected for the tests.

The artificial soil (SAT) used in the escape and mortality tests consisted of dry‐sieved fine sand (70%), kaolin powder (20%), and coconut fiber powder (10%). This soil composition was proposed by the OECD [36] for carrying out ecotoxicological tests with earthworms.

The escape and mortality tests used two replicates with 10 earthworms for each treatment.

#### 2.4.2 Escape Test

The escape test with *E. fetida* was carried out by NBR ISO 17.512‐1 [37].

Rectangular polypropylene containers were used, with a height of 115 mm and dimensions of 175 × 132 mm, and perforated lids to allow ventilation. Before the test, a removable plastic divider separated these containers in half. On each side of the container, 300 g of SAT was placed. Distilled water was used as a negative control, and boric acid was used as a positive control at a ratio of 750 mg to 1 kg of SAT soil, according to Nascimento et al. [17].

For each treatment, one side of the container holds SAT soil and distilled water, while the other side contained SAT soil and boric acid solution or SAT soil and the concentration of interest. For each side of the containers, the humidity was adjusted, in which 100 mL of the treatment solution was used for 600 g of soil.

After preparing the containers with their respective solutions, the plastic divider was removed, and 10 earthworms were placed on the dividing line between the two sides. The containers were then left in the dark for 48 h. After this time, the plastic dividers were replaced, separating the control soil from the treated soil, and the number of earthworms on each side of the container was counted. When replacing the divider, if any of the animals were cut, the side of the container on which most of the body remained was considered, according to Candello et al. [38].

A double negative control was carried out, in which both sections of the container hold SAT and distilled water. This condition is necessary to validate the escape test since the organisms are expected to be evenly distributed in the containers due to the absence of contaminants. In addition, the number of dead earthworms during exposure to the treatments was analyzed and must be less than 10% in each container.

The data were analyzed as a percentage of escape, according to Equation (7).

(7) $$\%\text{Escape}=\left[\frac{\left(\mathrm{nC}-\mathrm{nT}\right)}{N}\right]\times 100$$

where % is the percentage of escape, *nC* the number of earthworms found in section B (control soil), *nT* the number of earthworms found in section A (test soil), and *N* the total number of earthworms (sum of the two repetitions).

Based on the results, if the earthworms prefer the soil treated with the concentrations of interest, it is characterized as a negative response, with 0% escape. The soil with the treatments of interest is considered repellent when 40% (or more) of the exposed organisms prefer the control soil.

#### 2.4.3 Mortality Test

The mortality test on *E. fetida* was carried out by ABNT NBR 15537 [39], with modifications by Santo et al. [28].

Initially, rectangular polypropylene containers were used, with a height of 115 mm and dimensions of 175 × 132 mm, in which 600 g of SAT and 8 g of cattle manure were placed, plus distilled water up to 60% of the soil's maximum retention capacity. Ten earthworms were placed in each container, which were then covered with perforated plastic lids to allow ventilation. The containers were placed in a BOD oven for the animals to acclimatize at a temperature of 22°C ± 2°C, set to 16 h of light and 8 h of dark for 7 days.

After the acclimatization period, the animals were transferred to other previously prepared containers, which contained 600 g of SAT and 8 g of cattle manure, plus distilled water (negative control) or boric acid in the proportion of 750 mg/kg of SAT (positive control) or with concentrations of interest, up to 60% of the soil's maximum retention capacity (100 mL of water or positive control or concentrations). The containers were immediately placed in a BOD oven, programmed with the same temperature and light/dark cycle as the acclimatization period, where they remained for 14 days.

On the seventh day of incubation, the containers with earthworms were weighed, and where necessary, the humidity was corrected with their respective treatments. At this point, 8 g of manure was added to each container. After 14 days of incubation, the number of dead earthworms in each container was counted. The contents of the containers were poured into a plastic tray, and a mechanical stimulus was applied to the earthworms to ensure the animals were dead.

The data was expressed as a percentage of mortality (Equation 8):

(8) $$\%\text{Mortality}=\left[\frac{\mathrm{nM}}{N}\right]\times 100$$

where % is the percentage of mortality, *nM* the number of dead earthworms, and *N* the total number of earthworms.

### 2.5 Enzymatic Analysis of *A. cepa* Bulb Roots and *E. fetida*

#### 2.5.1 Preparation of Roots and Earthworms for Enzymatic Analysis

In plants, 50 mg of root meristems from each bulb (obtained in 2.4) were macerated in 1 mL of HCl (38%) and 2 mL of diethylenetriaminepentaacetic acid (5 mM) and centrifuged at 4000 rpm for 15 min to obtain the enzyme extracts.

In earthworms, the individuals that survived the mortality test (item 2.4.3) from each repetition were crushed individually and homogenized at 10 000 rpm with an Ultra‐Turrax crusher for 60 s in 10 mL of balanced salt solution (LBSS) prepared according to Hendawi et al. [40]. The homogenates were centrifuged for 15 min at 4000 rpm to obtain the enzyme extracts.

The enzyme extracts obtained were analyzed in a UV–Vis spectrophotometer for modulation of the enzymes catalase (CAT), ascorbate peroxidase (APX), guaiacol peroxidase (GPOX), and superoxide dismutase (SOD).

#### 2.5.2 Enzymatic Analysis

CAT analysis was based on Kraus et al. [41] with adaptations by Azevedo et al. [42]. 2.5 mL of sodium phosphate buffer solution (pH 7.8) was added to 100 μL of extract. For reading at 240 nm, 1 mL of 1 mM hydrogen peroxide (H_2_O_2_) was added to the samples. The extinction coefficient for the calculation was 2.8 M/cm, and the results were expressed in μmol/min/μg of protein (Equation 9).

(9) $$U=\frac{\left[\frac{\left(\frac{A}{t}\right)}{E}\right]\times V_{e}\times \mathrm{DF}}{P}$$

where *U* is the enzyme unit, *A* the measured absorbance, *t* the analysis time, *E* the extinction coefficient, *V*_*e*_ the enzyme volume, DF the dilution factor, and *P* the protein obtained from the mass of roots used.

APX analysis was based on Zhu et al. [43]. 2.5 mL of sodium phosphate buffer solution, 500 μL of 0.25 mM ascorbic acid, and 1 mL of 1 mM H_2_O_2_ were added to the extracts. The reading was carried out at 290 nm. The extinction coefficient was 2.8 M/cm; the results were expressed in μmol/min/μg of protein (Equation 9).

GPOX analysis was based on Matsuno and Uritani [44]. To 300 μL of extract, 2.5 mL of sodium phosphate buffer was added, 250 μL of 0.1 M citric acid, and 250 μL of 0.5% guaiacol. Then 250 μL of 1 mM H_2_O_2_ was added to the mixtures, which were stirred in a vortex and placed in an oven at 30°C for 15 min. The samples were then placed in an ice bath for 10 min, and 250 μL of 2% sodium metabisulphite was added. The samples were read at 450 nm. The extinction coefficient was 26.6; the results were expressed in μmol/min/μg of protein (Equation 9).

SOD analysis was based on Sun et al. [45]. The samples were prepared in duplicate, where half were kept under 80 W fluorescent light for 20 min, and the other half were kept in the dark. In 200 μL of the aliquots, 0.8 mL of sodium phosphate buffer and 500 μL of 0.1 mM ethylenediaminetetraacetic acid (EDTA) were added, as well as 500 μL of methionine, 500 μL of nitro blue tetrazolium chloride (NBT), and 200 μL of riboflavin. The samples were read at 560 nm, and the results were expressed in U of protein (Equation 10).

(10) $$\mathrm{SOD}=\frac{\left(\frac{B_{l}-s_{l}}{B_{l}}\right)-\left(\frac{B_{e}-s_{e}}{B_{e}}\right)}{50}$$

where *B*_*l*_ is the absorbance of the blank kept in the light prepared without the enzyme extract, *s*_*l*_ the absorbance of the sample kept in the light, *B*_*e*_ the absorbance of the blank kept in the dark, and *s*_*e*_ the absorbance of the sample kept in the dark. The quotient 50 represents the amount of enzyme required to inhibit 50% of the photoreduction of NBT.

### 2.6 Analysis of Antioxidant Activity (DPPH), Phenolic Content (Folin–Ciocalteu), and Lipid Peroxidation (TBARs) in Root Meristems and Earthworms

#### 2.6.1 Sample Preparation

In plants, 50 mg of root meristems from each bulb repetition (obtained in 2.4) was centrifuged in distilled water for 15 min at 4000 rpm to obtain homogenates (supernatant).

In animals, earthworms that survived the mortality test from each repetition were ground individually using an Ultra‐Turrax grinder at 10 000 rpm for 60 s in LBSS solution and centrifuged for 15 min at 4000 rpm to obtain homogenates.

#### 2.6.2 DPPH Assay on Roots

Antioxidant activity was assessed according to Unalan et al. [46], where 250 μL of 0.00316% (m/V) DPPH solution was added to 50 μL of homogenate. The mixture was then left to stand without light for 30 min. After this time, the mixtures were evaluated spectrophotometrically at 515 nm. Equation (11) was used to determine the antioxidant activity.

(11) $$A\left(\%\right)=\frac{A_{c}-A_{s}}{A_{c}}\times 100$$

where AA% is the antioxidant activity, *A*_*c*_ the absorbance of the DPPH solution without the sample, and *A*_*s*_ is the absorbance of the sample with DPPH.

#### 2.6.3 Folin–Ciocalteu (FC) Assay on Roots and Earthworms

The Carmona‐Hernandez [47] protocol was used for FC analysis. In 50 μL of homogenate, 100 μL of FC (0.0288 g of phosphotungstic acid and 0.0182 g of phosphomolybdic acid dissolved in 5 mL of methyl alcohol), 50 μL of ethanol, and 50 μL of distilled water were added. The mixtures were left in the dark for 10 min. Then, 50 μL of saturated sodium bicarbonate solution was added to the mixtures, which remained without light for 50 min. They were then evaluated spectrophotometrically at 745 nm.

#### 2.6.4 TBARs Assay on Roots and Earthworms

Lipid peroxidation was analyzed according to Papastergiadis et al. [48]. Two hundred and fifty microliters of TBARs solution (46 mM) was added to 50 μL of homogenate. The mixture was kept in a heat bath at 90°C for 35 min. After cooling, it was analyzed spectrophotometrically at 532 nm.

### 2.7 Statistical Analysis

The data on phytotoxicity, cytotoxicity, genotoxicity, antioxidant enzymes, DPPH, FC, and TBARs were subjected to Kruskal–Wallis analysis of variance followed by Dunn's test (*p* ≤ 0.05) using RStudio software.

In the escape test, the mean data and standard deviation of the number of *E. fetida* found in each container section were analyzed using Fisher's one‐tailed test, using Action 6.2 software.

## 3 Results and Discussion

**FIGURE 1:**
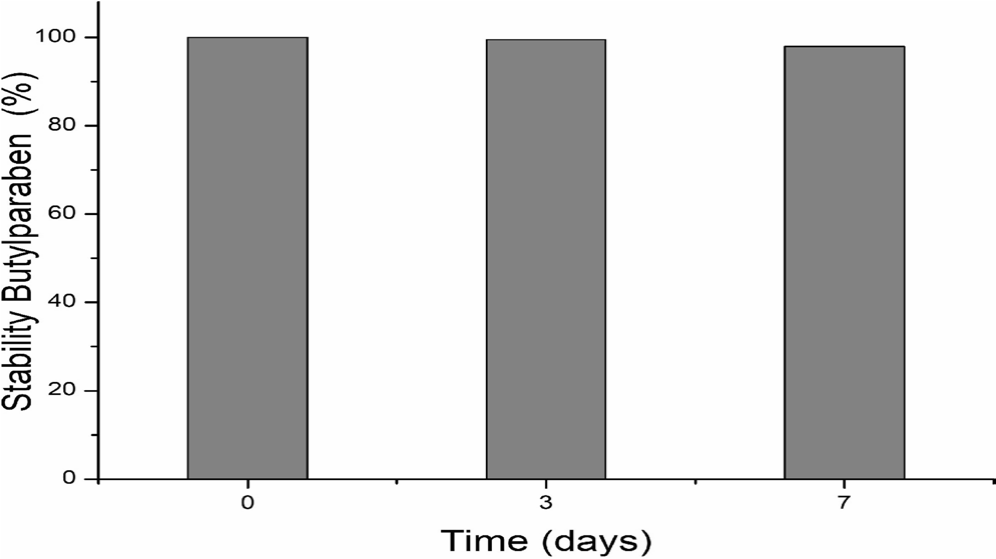
Stability of butylparaben in aqueous media for 7 days in the absence of light.

The stability of BuP was analyzed in an aqueous medium, without the presence of light and at room temperature, for 7 days (Figure 1). At time zero, the concentration was 100%. BuP was found to be stable throughout the analysis period.

### 3.1 Toxicity and Oxidative Stress in Seed Roots and in Onion Bulb Roots

Table 1 shows that all the concentrations of this paraben in *D. carota* , *A. cepa* , and *C. sativus* did not cause a significant reduction in seed germination. However, the four concentrations in carrot, onion, and cucumber caused a RGI of less than 0.8, which characterizes significant inhibition of root growth. The 10, 50, and 100 ng/L concentrations in the three plants caused a GI of between 50% and 80%, demonstrating moderate toxicity of BuP to seeds. The 500 ng/L concentration in the three vegetables caused a GI of less than 50%, demonstrating high toxicity with lethal potential. Therefore, it can be concluded that BuP caused significant phytotoxicity to the rootlets of *D. carota* , *A. cepa* , and *C. sativus* seeds.

**TABLE 1:** Phytotoxic potential of butylparaben in seeds of *Daucus carota* L., *Allium cepa* L., and *Cucumis sativus* L. based on the parameters seed Germination, Relative Growth Index, and Germination Index.

Butylparaben			
*Daucus carota* L.			
TR	G (%)	RGI/SD (%)	GI/SD (%)
Co	91	1	100
10 ng/L	85	0.67 ± 2.0*	63.3 ± 1.5*
50 ng/L	82	0.66 ± 1.5*	59.8 ± 1.5*
100 ng/L	79	0.66 ± 1.7*	57.4 ± 1.0*
500 ng/L	99	0.44 ± 1.5*	44.0 ± 1.0*

*Allium cepa* L.			
TR	G (%)	RGI/SD (%)	GI/SD (%)
Co	98	1	100
10 ng/L	96	0.57 ± 1.3*	56.5 ± 1.5*
50 ng/L	98	0.53 ± 2.0*	52.8 ± 1.0*
100 ng/L	94	0.54 ± 1.0*	52.7 ± 1.8*
500 ng/L	99	0.45 ± 1.5	40.0 ± 1.5*

*Cucumis sativus* L.			
TR	G (%)	RGI/SD (%)	GI/SD (%)
Co	97	1	100
10 ng/L	95	0.68 ± 2.0*	66.5 ± 2.0*
50 ng/L	93	0.65 ± 1.5*	61.7 ± 2.0*
100 ng/L	91	0.64 ± 1.3*	59.6 ± 1.5*
500 ng/L	99	0.43 ± 1.5*	42.3 ± 1.5*

*Note*: *Significant difference in relation to the control, according to Kruskal–Wallis H followed by Dunn's post hoc test (*p* ≤ 0.05).

Abbreviations: Co: control, G: seed germination, GI: Germination Index, RGI: Relative Growth Index, SD: standard deviation, TR: treatment.

In Table 2, the four concentrations of BuP were phytotoxic since they caused a significant reduction in root growth in *A. cepa* bulbs, corroborating the results of root growth inhibition in *D. carota* , *A. cepa* , and *C. sativus* seeds (Table 1). The 10, 50, and 100 ng/L concentrations in bulbs significantly inhibited cell division in the root meristems, proving cytotoxic. However, the mitotic indices obtained were above 50% of the Mitotic Index estimated for the control, unlike the 500 ng/L concentration, which showed a cell division index equal to 21.5%. It is known that mitotic indices below 50% cause sublethal effects, and below 22% can cause the death of the organism [49]. The mito‐inhibitory effect observed in bulb roots was dose‐dependent. It demonstrated that BuP can potentially cause cell cycle arrest in prophase by inducing damage to DNA synthesis and/or protein synthesis and/or DNA repair mechanisms.

**TABLE 2:** Analysis of the phytotoxic, cytotoxic, and genotoxic potential of butylparaben, at concentrations of 10, 50, 100, and 500 ng/L, in *Allium cepa* L. bulb roots, based on the parameters Average Root Length, Mitotic Index, and Cell Alteration Index.

TR	ARL (%)	MI/SD (%)	CAI/SD (%)
Co	100	100	0.1 ± 0.9
10 ng/L	53.5 ± 2.0*	61.7 ± 1.5*	3.5 ± 1.5*
50 ng/L	50.4 ± 1.5*	58.7 ± 1.7*	4.0 ± 1.8*
100 ng/L	51.7 ± 1.5*	50.4 ± 1.5*	5.7 ± 1.2*
500 ng/L	33.8 ± 1.9*	21.5 ± 1.0*	1.5 ± 1.0*

*Note*: For ARL and MI, the data is expressed as a percentage of the Co values. *Significant difference in relation to the control, according to Kruskal‐Wallis H followed by Dunn's post hoc test (*p* ≤ 0.05).

Abbreviations: ARL: average root length, CAI: Cell Alteration Index, Co: control, MI: Mitotic Index, SD: standard deviation, TR: treatment.

**FIGURE 2:**
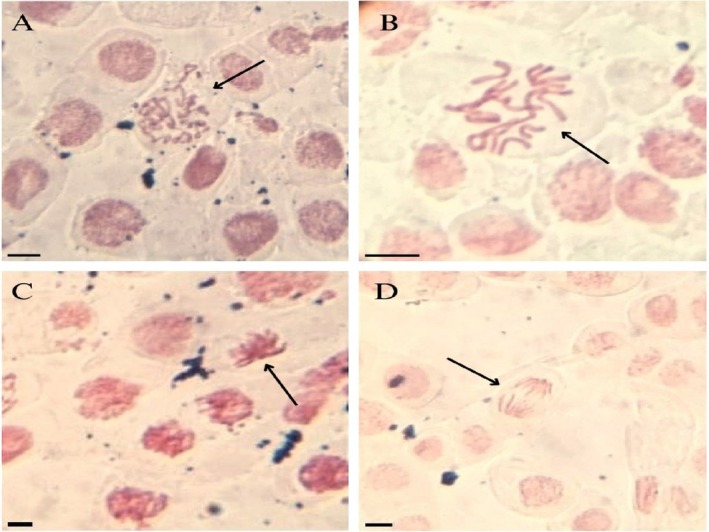
Cellular alterations observed in root meristems of *Allium cepa* L. bulbs exposed to butylparaben at concentrations of 10, 50, 100, and 500 ng/L for 5 days. (A) chromosome disorganization in prophase, (B) chromosome disorganization in metaphase, (C) viscous metaphase, and (D) anaphase bridge. Bar: 10 μm.

Table 2 also shows that BuP, at all concentrations, induced significant indices of cellular changes in root meristems (Figure 2). The cellular alterations observed were chromosome disarray in prophase and metaphase (Figure 2A,B), and chromosome bridge in anaphase (Figure 2D), caused by disturbances in the organization and/or functioning of the mitotic spindle, characterizing BuP under these conditions of analysis as a genotoxic compound with aneugenic action. According to Beijora et al. [50], mitotic spindle alterations in meristems are highly damaging to plants as they cause chromosome loss and the formation of aneuploid cells, compromising the development and functioning of organs. In addition, the four concentrations of this paraben induced the formation of slimy metaphases in the roots (Figure 2C). According to Bakare et al. [51], viscosity in chromosomes indicates that the pollutant has affected the organization of chromatin by causing an imbalance in the distribution of histones during chromosome condensation and is characterized as a highly lethal alteration to cells. It can be seen that BuP at 500 ng/L had the lowest rate of cellular alterations of the concentrations evaluated due to the drastic reduction in cell division caused by this concentration in root meristems.

**FIGURE 3:**
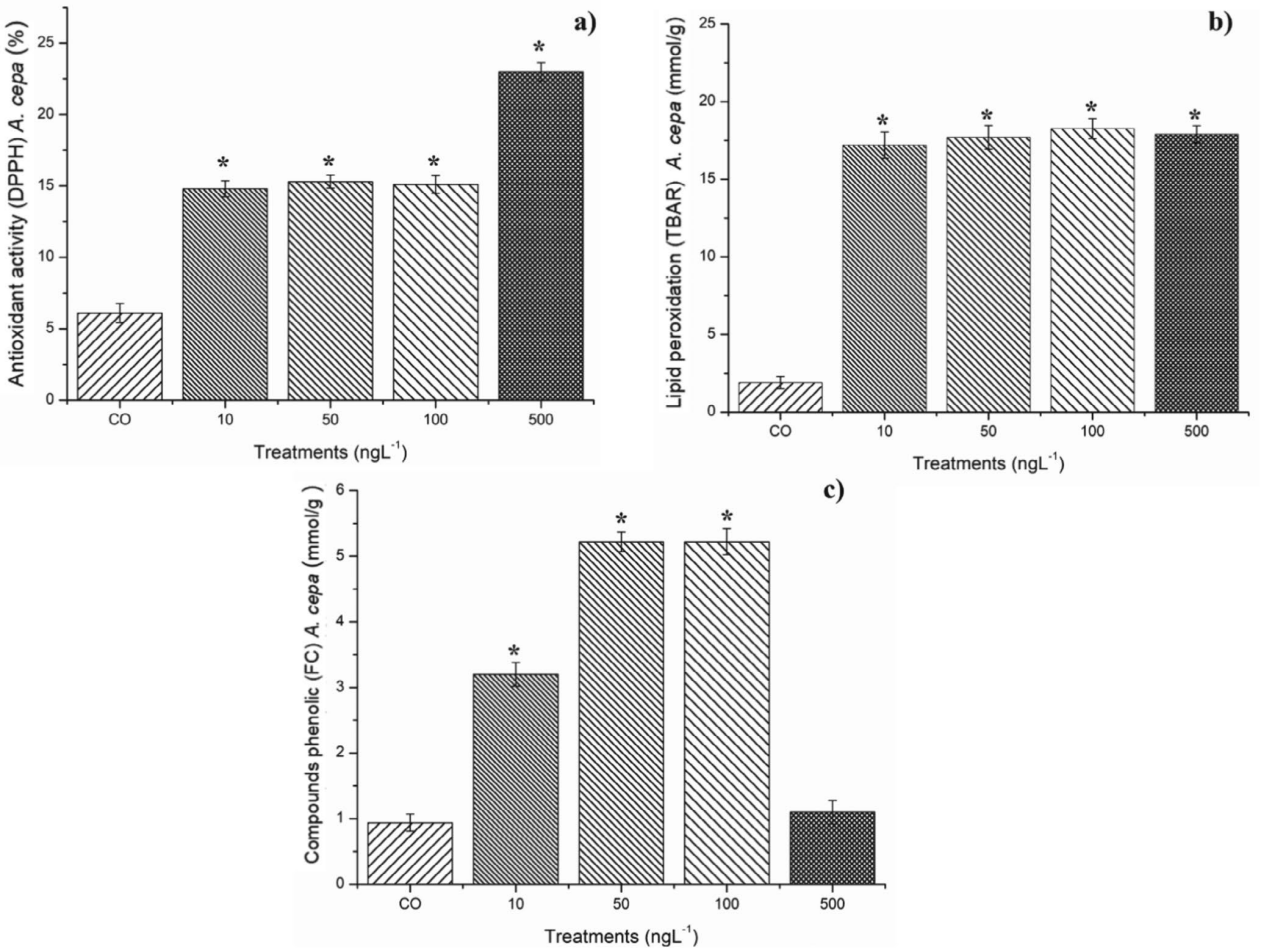
(a) Antioxidant activity (DPPH), (b) lipid peroxidation (TBAR), and (c) concentration of phenolic compounds (FC) in *A. cepa* bulb roots exposed to butylparaben at concentrations of 10, 50, 100, and 500 ng/L. Significant difference in relation to the control according to Kruskal–Wallis H followed by Dunn's post hoc test (*p* ≤ 0.05).

Figure 3a shows that the four concentrations of BuP caused a significant increase in antioxidant activity in the meristems, in which the 500 ng/L concentration caused the most significant increase. In addition, the 10, 50, and 100 ng/L concentrations significantly increased the concentration of phenolic compounds in the cells (Figure 3c), reiterating the harmfulness of BuP to root meristems, which activated the cellular defense system through the production of phytochemicals (Figure 3a) in order to preserve root growth, but without success (Tables 1 and 2, Figure 2). Furthermore, the 500 ng/L concentration, compared to the control, caused only a slight increase in phenolic compounds (Figure 3c), demonstrating that BuP raised the levels of oxidizing radicals to the point of inhibiting part of the cellular antioxidant system.

**FIGURE 4:**
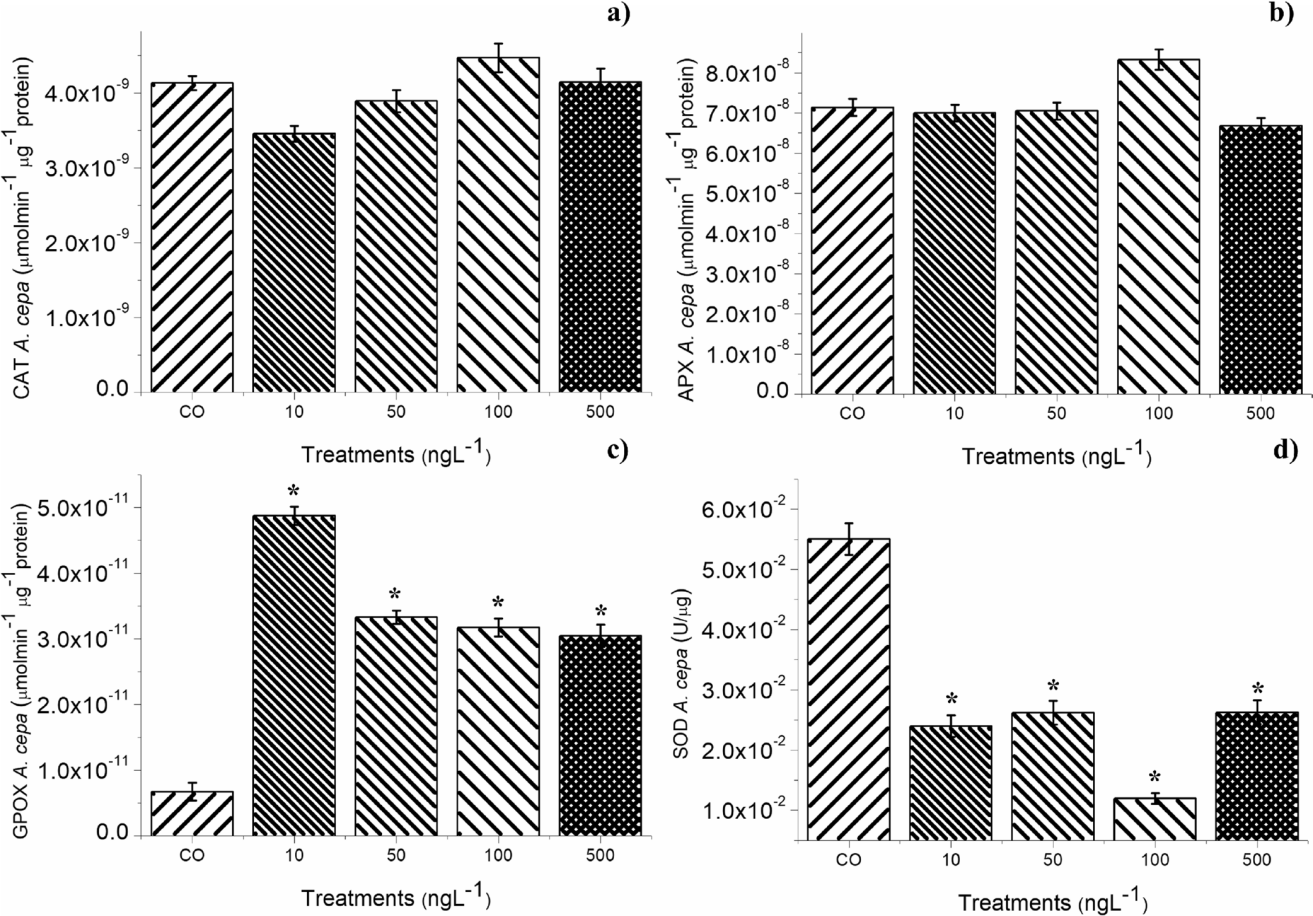
Modulations of the enzymes: (a) catalase (CAT), (b) ascorbate peroxidase (APX), (c) guaiacol peroxidase (GPOX), and (d) superoxide dismutase (SOD) in bulb roots of *Allium cepa* L. exposed to butylparaben at concentrations of 10, 50, 100, and 500 ng/L. Significant difference in relation to the control according to Kruskal–Wallis H followed by Dunn's post hoc test (*p* ≤ 0.05).

The antioxidant enzymes CAT, APX, SOD, and GPOX were analyzed in *A. cepa* bulb roots (Figure 4). These enzymes maintain the homeostasis of cell function, such as DNA and protein synthesis, and cell division, in tissues with intense cell proliferation [17, 52], such as the root meristems evaluated in this study. Figure 4 shows that the roots exposed to BuP at concentrations of 10, 50, 100, and 500 ng/L did not significantly alter the modulation of CAT and APX (Figure 4a,b). However, they did cause a significant reduction in SOD activity (Figure 4d), which increased the levels of superoxide radicals in the meristems. Furthermore, all four concentrations caused a significant increase in the expression of GPOX (Figure 4c). The activation of this enzyme suggests damage to the plasma membrane, which was confirmed by the TBARs results (Figure 3b), which showed that all concentrations caused lipid peroxidation in the meristematic cells. Among the main radicals resulting from lipid peroxidation are hydroxyls, hydroxypropyl ketones, aldehydes, and carboxylic acids, which are highly reactive to cellular proteins [49].

Superoxide radicals and those originating from lipid peroxidation cause severe and often irreversible cell damage, such as denaturation of proteins linked to DNA replication, protein synthesis, and DNA repair, as well as those responsible for the assembly and functioning of the mitotic spindle [50, 53, 54]. Thus, the accumulation of oxidizing radicals in the meristems exposed to BuP explains the significant increase in antioxidant activity in the cells (Figure 3a,c), but this was not sufficient to contain the cell cycle arrest in interphase and the induction of cellular alterations (Table 2 and Figure 2), causing a significant reduction in root growth in seeds and bulbs (Tables 1 and 2).

Corroborating the cell proliferation results obtained for plants, Li et al. [6], studying zebrafish larvae exposed to BuP at concentrations in the mg/Kg range, observed a significant increase in superoxide and hydroxyl radicals in the neural crest cells of these organisms, which caused a significant reduction in cell proliferation. Guzel et al. [55], studying human peripheral lymphocytes exposed to BuP at concentrations in μg/L, found a reduction in cell division and high rates of cellular alterations in the cells. In addition, Todorovac et al. [56] observed that BuP caused a reduction in cell division and cellular alterations in *A. cepa* roots, corroborating the results obtained in the present study. However, the concentrations evaluated by these authors were on the mg/L scale, very high when compared to the real concentrations of this compound in the environment, which are generally on the ng scale. However, the results of cytotoxicity and genotoxicity for BuP, according to Todorovac et al. [56], emphasize the harmfulness of this compound to plants since, in the present study, similar toxicity was obtained, but at concentrations thousands of times lower. Furthermore, based on the results obtained here and the results of the three studies mentioned above, it is possible to infer that BuP has a direct action on the genetic material of cells.

**FIGURE 5:**
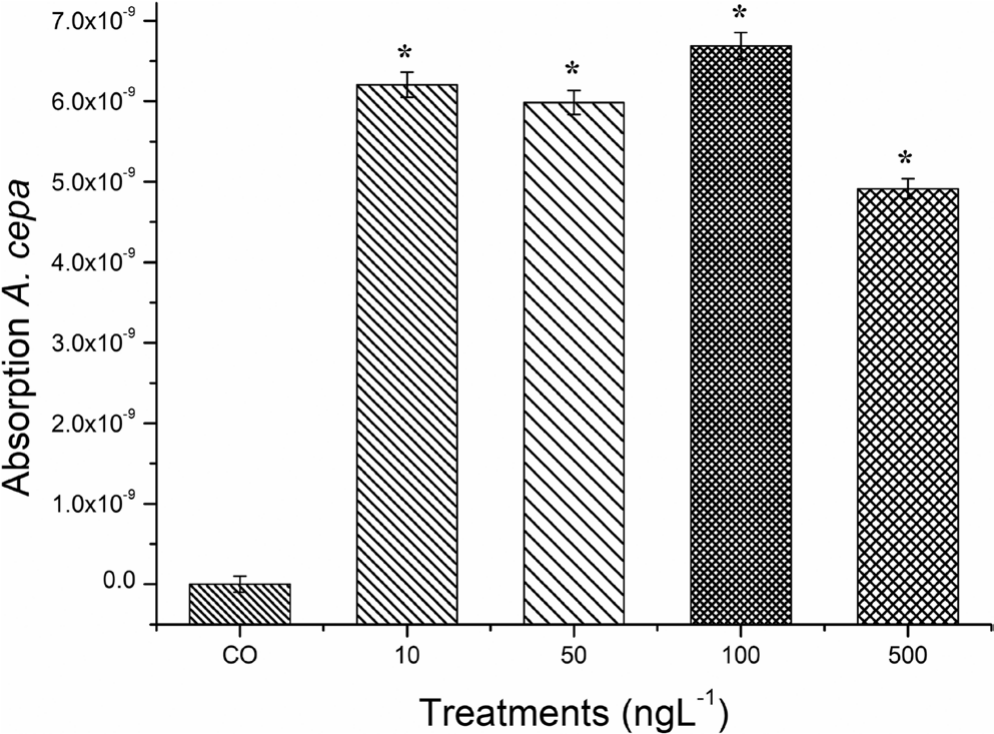
Absorption of butylparaben by roots of *Allium cepa* L. after 5 days of exposure to concentrations of 10, 50, 100, and 500 ng/L of this compound. *Significant difference in relation to the control according to Kruskal–Wallis H followed by Dunn's post hoc test (*p* ≤ 0.05).

Therefore, based on the biomarkers evaluated in plants, which were root growth in seeds and bulbs, cell proliferation and cellular changes in root meristems of bulbs, and enzymatic and non‐enzymatic changes in cellular functioning of bulb roots, it is concluded that the four concentrations of BuP triggered oxidative stress in cells (Figures 3 and 4), since they mainly increased the concentration of superoxide radicals and of radicals resulting from lipid peroxidation in cells, which caused a significant reduction in cell division, as well as a significant frequency of mitotic spindle changes in the root meristems (Table 2), causing a significant reduction in root growth in seeds of *D. carota* , *A. cepa* , and *C. sativus* (Table 1), and in the roots of bulbs of *A. cepa* (Table 2). It is noteworthy that the concentration of 500 ng/L was the most harmful of the concentrations evaluated. Furthermore, based on the results of absorption of this compound by bulb roots (Figure 5), it can be seen that this concentration was the least absorbed, further highlighting its toxicological potential compared to the other concentrations evaluated.

According to Sharma et al. [57] and Gill et al. [58], systemic and cellular toxicity resulting from oxidative stress, such as those triggered by BuP in plants (Tables 1 and 2, Figure 2), is among the leading causes of loss of productivity in different crops around the world because it negatively influences cellular and physiological mechanisms in plants. In addition to the yield factor, leaching from BuP‐contaminated soils also threatens the survival of spontaneous plants and the organisms that depend on them, representing an environmental risk.

### 3.2 Behavioral Effect, Toxicity, and Oxidative Stress in Earthworms

**FIGURE 6:**
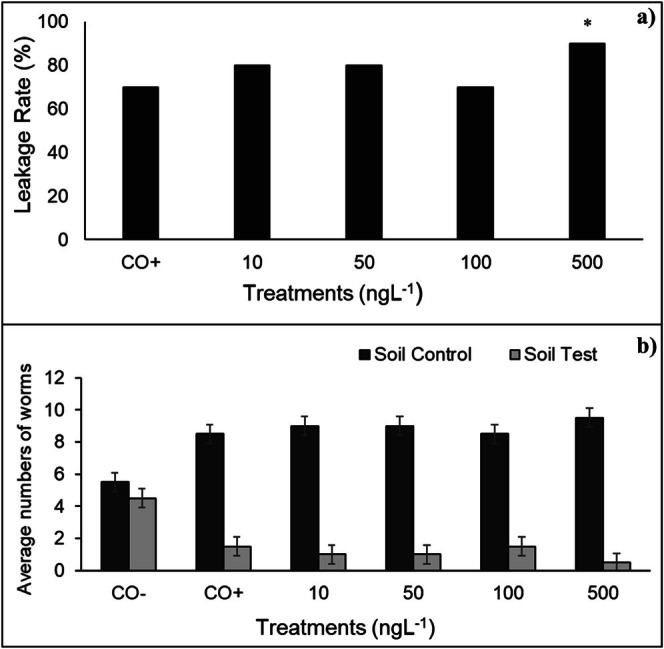
Percentage escape of *Eisenia fetida* Sav. exposed to the positive control (CO+) and BuP at concentrations of 10, 50, 100, and 500 ng/L (a) and percentage of earthworms in the control soil and soil with BuP concentrations (b). *Significant difference to the control, according to one‐tailed Fischer's Exact Test (*α* = 0.05; *p* < 0.04).

In animals, the influence of BuP on the behavior of *E. fetida* earthworms was evaluated using the escape test (Figure 6). This test is based on the ability of earthworms to perceive xenobiotics through chemoreceptor tubercles in their body, located in the protostome and mouth region [59]. These structures, combined with their ability to move, allow these animals to choose whether or not to occupy soil contaminated with pollutants [60]. Figure 6a shows that the 10, 50, 100, and 500 ng/L concentrations of BuP in SAT soil caused 80%, 80%, 70%, and 90% escape, respectively. For the 500 ng/L concentration, the distribution of organisms was statistically different from the experiment's negative control, according to Fisher's Exact Test. Validating the escape result for this paraben, the distribution of earthworms in the dual control soil was 55% and 45% between the two sides (Figure 6b).

According to ISO [61], the percentage of escape between 40% and 80%, as observed for the 10, 50, and 100 ng/L concentrations of BuP (Figure 6a), characterizes an expressive repellent effect of the pollutant on earthworms, while above 80%, as observed for the 500 ng/L concentration, characterizes a high repellency of the pollutant to these animals with potential loss of habitat. According to Wijayawardena et al. [62], the repellency of these animals to xenobiotics in the soil can significantly impact the survival of different edaphic species since earthworms make up 80% of soil biomass and are fundamental for the decomposition of organic matter and aeration in this environment, as well as being essential to the terrestrial trophic chain.

**FIGURE 7:**
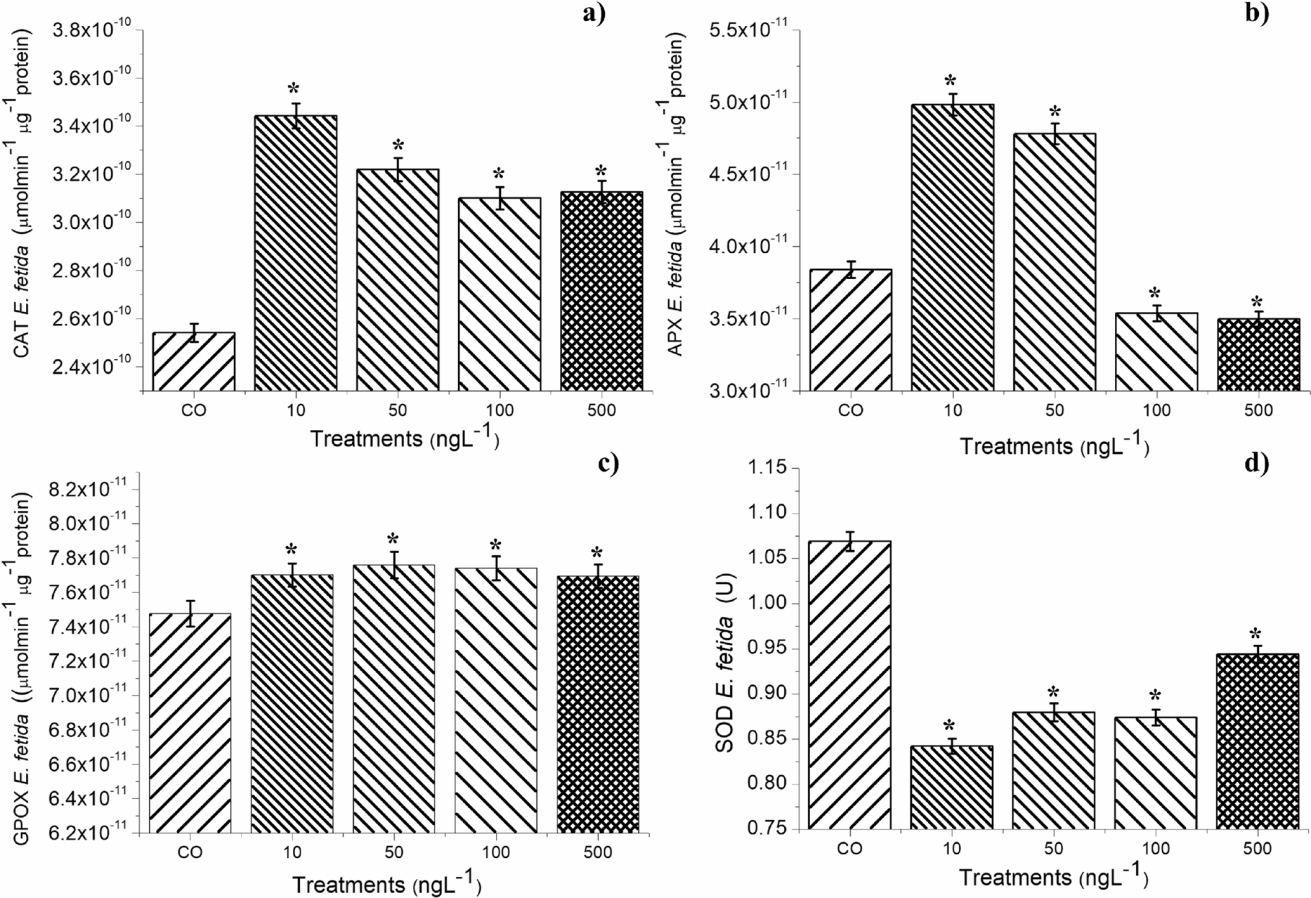
Modulation of the enzymes: (a) CAT (catalase), (b) APX (ascorbate peroxidase), (c) GPOX (guaiacol peroxidase), and (d) SOD (superoxide dismutase) in *Eisenia fetida* Sav. earthworms after 14 days of exposure to butylparaben at concentrations of 10, 50, 100, and 500 ng/L. *Significant difference in relation to the control according to Kruskal–Wallis H followed by Dunn's post hoc test (*p* ≤ 0.05).

**FIGURE 8:**
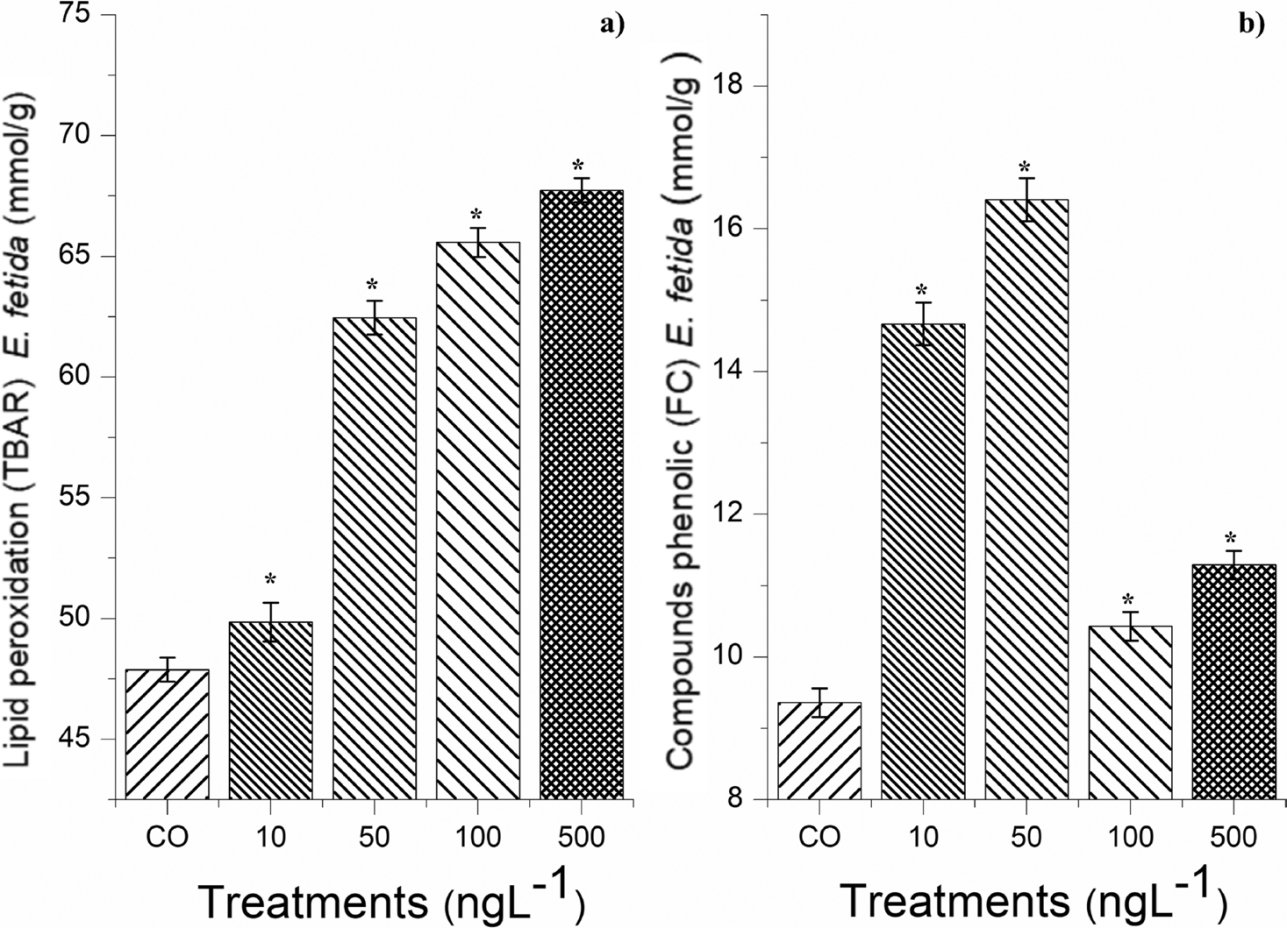
(a) Lipid peroxidation (TBAR) and (b) concentration of phenolic compounds (FC) in *Eisenia fetida* Sav. earthworms after 14 days of exposure to butylparaben at concentrations of 10, 50, 100, and 500 ng/L. *Significant difference in relation to the control according to Kruskal–Wallis H followed by Dunn's post hoc test (*p* ≤ 0.05).

The four concentrations of BuP did not cause mortality in *E. fetida* after 14 days of exposure. However, after analyzing the enzymes in the animals (Figure 7), it was found that all the concentrations caused a significant reduction in SOD (Figure 7d), which consequently increased the concentration of superoxide radicals in the tissues of these organisms. SOD activity is fundamental in earthworms, and its reduction/inhibition generates instability in the cellular defense system, triggering lipid peroxidation in the tissues [6]. All concentrations caused a significant increase in CAT (Figure 7a), and the 10 and 50 ng/L concentrations caused a significant increase in APX (Figure 7b), showing that for these two concentrations, although BuP caused an increase in the concentration of hydrogen peroxide in the cells, it was effectively degraded. However, at concentrations of 100 and 500 ng/L, APX was expressly inhibited, allowing peroxide to accumulate in the tissues and, therefore, hydroxyl radicals. Corroborating these results, Figure 8b shows a significant reduction in the production of phenolic compounds in earthworms exposed to 100 and 500 ng/L concentrations, demonstrating that the cells were disarmed against oxidative radicals. Furthermore, BuP in all four concentrations caused activation of GPOX (Figure 7c), which, based on the TBARs results (Figure 8a), shows that this compound triggered lipid peroxidation in the tissues.

Therefore, BuP at the concentrations evaluated causes earthworms to escape, which can cause imbalance in the soil's micro and macrobiota. Furthermore, although the earthworms exposed to the four concentrations of this compound for 2 weeks did not die, it was found that BuP triggered significant oxidative stress in these animals. According to Santo et al. [28] and Nascimento et al. [17], superoxide radicals, hydroxyl radicals, and those originating from lipid peroxidation have a high potential to cause tissue damage in earthworms, which, when they do not cause death, drastically affect the survival of these organisms, significantly altering their ecological functions and, consequently, the homeostasis of the ecosystem in which they are inserted. Wijayawardena et al. [62] report that the possible sublethal effects of oxidative stress on these animals are inhibition of growth and development, loss of biomass, reduction in reproduction rate, inhibition of cell division, and intestinal and epidermal damage, which consequently make them weaker in the face of adverse environmental conditions.

Corroborating the oxidative stress results observed in earthworms, Nagar et al. [13] found that BuP, in μg/L, triggered oxidative stress, toxicity, and endocrine disruption in the soil nematode *Caenorhabditis elegans* . Aydemir et al. [63] demonstrated that BuP, in mg/Kg, triggered significant oxidative stress in the spleen, liver, and kidneys of Wistar rats after 28 days of exposure. In addition, Silva et al. [64] found that this compound, in μg/L, significantly increased the concentration of hydroxyl radicals in the liver and gills of *Oreochromis niloticus* L. fish after 6 and 12 days of exposure. Martins et al. [65] found that BuP drastically interfered with the enzymatic antioxidant system in the testes of Wistar rats, triggering significant genotoxicity. However, unlike the results with earthworms obtained here, Samaranghe et al. observed that BuP, in μg/L and up to 72 h of exposure, did not negatively affect the growth and reproduction of *E. fetida* . It should be noted that the exposure time considered in this study was acute, and no oxidative stress analysis was carried out on the worms to assess sublethal effects.

## 4 Conclusion

BuP in plants, after 5 days of exposure, caused oxidative stress in root meristems, which triggered a reduction in cell proliferation and the formation of cellular alterations, significantly reducing root growth in seeds and bulbs. The 500 ng/L concentration was the most harmful to plants.

In animals, soil contamination with BuP caused high evasion of earthworms within 48 h of exposure. This antimicrobial did not cause the death of earthworms after 14 days of exposure, but it triggered significant oxidative stress in their tissues. The 100 and 500 ng/L concentrations were the most harmful to the animals.

## Author Contributions

All authors contributed to the study conception and design. Material preparation, data collection, and analysis were performed by Lorena Maihury Santos Tsubouchi, Edson Araújo de Almeida, Diane Scapin, Anna Zhuolina Gomes Oliveira, Cassiano Aparecido de Souza, Diego Espirito Santo, Carmem Lúcia Henrich, Ana Elisa Maehashi, Gideã Taques Tractz, Osvaldo Valarini Junior, Regiane da Silva Gonzalez, Elisângela Dusman, and Ana Paula Peron. The final draft of the manuscript was written by Ana Paula Peron, with critical review for important intellectual content by Craig Allan Downs, Regiane da Silva Gonzalez, and Elisângela Dusman. All authors read and approved the final manuscript.

## Conflicts of Interest

The authors declare no conflicts of interest.

## Data Availability

Data sharing is not applicable to this article as no new data were created or analyzed in this study.
